# Engineering Ti_3_C_2_-MXene Surface Composition for Excellent Li^+^ Storage Performance

**DOI:** 10.3390/molecules29081731

**Published:** 2024-04-11

**Authors:** Minghua Chen, Qi Fan, Ping Yu, Ke Chen, Peng Li, Kun Liang

**Affiliations:** 1 School of Materials Science and Chemical Engineering, Ningbo University, Ningbo 315211, China; 2 Zhejiang Key Laboratory of Data-Driven High-Safety Energy Materials and Applications, Ningbo Key Laboratory of Special Energy Materials and Chemistry, Ningbo Institute of Materials Technology and Engineering, Chinese Academy of Sciences, Ningbo 315201, China; 3 University of Chinese Academy of Sciences, 19 A Yuquan Rd, Shijingshan District, Beijing 100049, China; 4 School of Electronic and Information Engineering, Ningbo University of Technology, Ningbo 315211, China; 5 Qianwan Institute of CNITECH, Ningbo 315336, China

**Keywords:** MXenes, CVD, heterostructure, Li-ion battery, energy storage

## Abstract

Exploiting novel materials with high specific capacities is crucial for the progress of advanced energy storage devices. Intentionally constructing functional heterostructures based on a variety of two-dimensional (2D) substances proves to be an extremely efficient method for capitalizing on the shared benefits of these materials. By elaborately designing the structure, a greatly escalated steadiness can be achieved throughout electrochemical cycles, along with boosted electron transfer kinetics. In this study, chemical vapor deposition (CVD) was utilized to alter the surface composition of multilayer Ti_3_C_2_T*_x_* MXene, contributing to contriving various layered heterostructure materials through a precise adjustment of the reaction temperature. The optimal composite materials at a reaction temperature of 500 °C (defined as MX500), incorporating MXene as the conductive substrate, exhibited outstanding stability and high coulombic efficiency during electrochemical cycling. Meanwhile, the reactive sites are increased by using TiS_2_ and TiO_2_ at the heterogeneous interfaces, which sustains a specific capacity of 449 mAh g^−1^ after 200 cycles at a current density of 0.1 A g^−1^ and further demonstrates their exceptional electrochemical characteristics. Additionally, the noted pseudocapacitive properties, like MXene materials, further highlight the diverse capabilities of intuitive material design. This study illuminates the complex details of surface modification in multilayer MXene and offers a crucial understanding of the strategic creation of heterostructures, significantly impacting sophisticated electrochemical applications.

## 1. Introduction

Rechargeable energy storage technologies such as lithium-ion batteries have been considered the most promising candidates for solving the spreading global energy crisis [1,2]. While graphite is widely used as an anode material in lithium-ion batteries, its limited theoretical capacity makes it impossible to scale in large-scale applications [3]. Hence, there is a relentless demand for developing advanced anode materials with ultra-high capacity and extraordinary cyclic performance. Two-dimensional materials are attracting enormous attention owing to their unparalleled properties, consisting of a larger family of graphene [4], transition metal dichalcogenides (TMDs) [5], MXenes [6], and so on [7]. Intriguingly, unique structures with several atomic layers for significant ion storage and charge transfer bestow them with remarkable performance compared with other electrode materials.

In this context, the extensively studied transition metal dichalcogenide, like titanium disulfide (TiS_2_), displays a stratified composition with van der Waals gaps, assisting in the expedient intercalation and deintercalation of Li^+^ ions during the charge–discharge process [8]. Furthermore, TiS_2_ provides abundant active sites for ion storage and is thus conducive to a remarkable theoretical capacity, standing out amongst numerous 2D materials [9]. Nevertheless, recent studies demonstrate that its significance has slowly waned, which can undoubtedly be ascribed to its inferior cyclic stability and irreversible phase transitions, resulting from the inferior electrochemical activity between neighboring layers of TMDs and their unstable crystal structures [10,11]. It has been reported that the intercalated alkali metal ions like Li^+^ lead to the transversal gliding of an S plane and, thus, to the unexpected phase transitions from the 2H to the 1T phase [12].

Prior studies have demonstrated that the deliberate construction of heterostructures in a two-dimensional context affords a means to precisely modulate the physical and chemical properties of materials [13,14]. Constructing heterostructures with varying energy levels allows for the modulation of the internal electric field [15]. The distinct benefits of heterostructures stem directly from the distinctive microstructure at the heterogeneous interfaces. Upon the amalgamation of the two constituent components, charges undergo redistribution along these interfaces. Heterostructure materials deliberately incorporate an increased number of heterointerfaces, thereby maximizing the advantages of alterations within the heterostructure materials.

Two-dimensional transition metal carbonitrides, noted as MXenes, have been extensively studied since their discovery in 2011 by Gogotsi and colleagues [16]. Of note, MXenes exhibit a unique structure and composition, aligning with the P63/mmc crystal symmetry. Defined by the formula M_n+1_X_n_T*_x_*, M stands for transition metals such as Ti, V, and Nb, among others [17,18,19], and X represents carbon, nitrogen, or a combination of both. The surface termination, denoted by T*_x_*, is intricately linked to the employed etching method, featuring common compositions such as -F, -O, -OH, and -Cl [20]. Notably, recent studies have unveiled additional terminal groups, including -S, -P, and -Te [21,22]. More than 40 kinds of distinct stoichiometric MXenes have been reported [18], and this rare combination of elements and terminations defines the fascinating structure of MXenes, which is conducive to their diverse properties and applications. MXene has excellent electrical and mechanical properties and can be used as a conductive substrate to improve electron transport and the stability of electrodes [23,24,25,26,27,28].

Despite the high praise for MXenes, especially in the field of energy storage, due to their high electrical conductivity, large specific surface area, and unique pseudocapacitive characteristics [29], there are still important challenges, including restacking issues caused by strong van der Waals forces between MXenes nanosheets and their irreversible capacity during the first discharge process from the formation of solid electrolyte interface (SEI) films [30,31,32]. Introducing carbon nanotubes, silicon, metal sulfides, or metal oxides into the layers of MXenes can alleviate the stacking problem of MXene; meanwhile, MXenes can significantly improve the stability of intercalated materials during electrochemical cycling [33,34,35,36,37].

**Figure 1:**
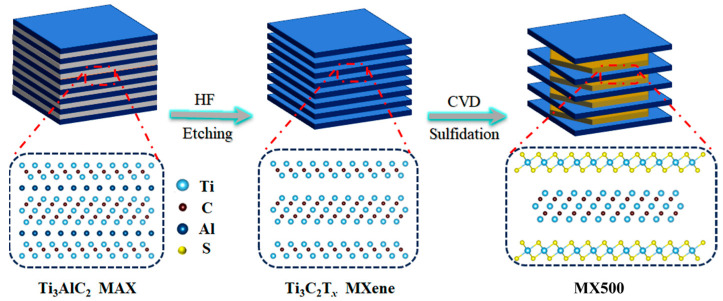
Schematic diagram of sulfidation of Ti_3_C_2_T*_x_* for preparation of MX500.

In this study, we synthesized a series of heterostructure materials termed MX-n (*n* = 500, 600, 700, and 800, presenting CVD temperature), as shown in Figure 1. These materials were derived from MXene sulfidation at varying degrees. We transformed the multilayer MXene’s outer layers into TiS_2_ and a small amount of titanium dioxide (TiO_2_), resulting in a “TiS_2_@TiO_2_@MXene” sandwich-like structure. Controlled temperature variations achieved different sulfurization levels.

Remarkably, these materials deliver higher capacities and superior cycle performances. MXene served not only as a conductive substrate but also provided a transition metal atom for sulfidation, leading to an ideal heterostructure cyclability (delivering a capacity of 400 mAh g^−1^ at 0.2 A g^−1^ after 200 cycles). The in-situ formation of TiS_2_ and TiO_2_ improved interfacial connections and enriched active sites, contributing to the high capacity of 400 mAh g^−1^. Our study unveiled structural and composition evolution insights, elucidating an enhanced structure–performance relationship. Our study demonstrated the feasibility of enhancing the material performance by modifying MXenes, providing valuable directions for future research.

## 2. Results and Discussion

### 2.1. Morphology and Chemical Composition

**Figure 2:**
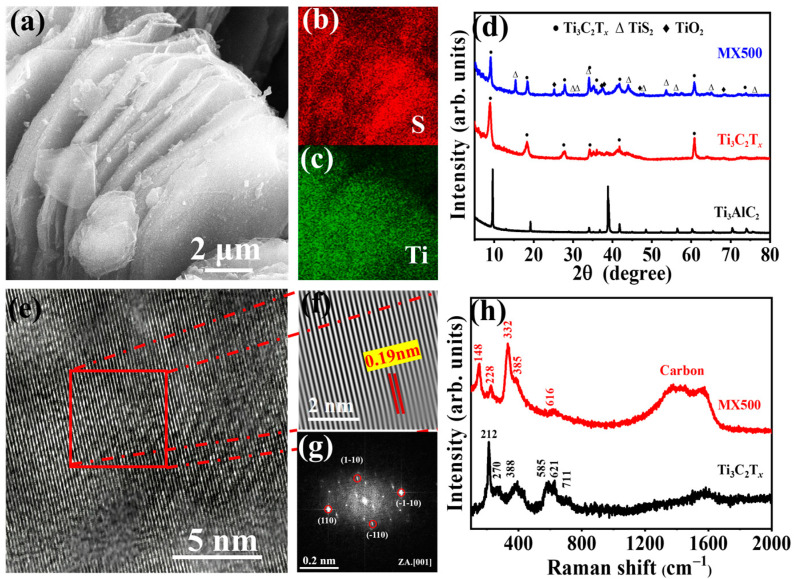
(**a**) The SEM image of MX500 and its corresponding element distribution with S in (**b**) the red area and Ti in (**c**) the green area; (**d**) XRD curves of Ti_3_AlC_2_, Ti_3_C2T_x_, and MX500; (**e**) the HRTEM image of MX500; (**f**) IFFT pattern of the selected area of (**e**); (**g**) FFT pattern of MX500; (**h**) Raman spectra curves of Ti_3_C_2_T_x_ and MX500.

The Ti_3_AlC_2_ MAX phase was etched by using a 10% HF solution. Obvious accordion-like structures were observed after etching (Figure S1a), which was the result of a vigorous reaction of aluminum (Al) in the MXene precursor material with concentrated hydrofluoric acid (HF). The EDS elemental distribution mappings showed that the surface terminations consist mainly of oxygen and fluorine (as shown in Figure S1b), and the EDS data (Table S1) indicated a dramatic decrease in aluminum elements. The phase and structure of the sample were detected by XRD. Compared to Ti_3_AlC_2_, the *(00l)* peaks of Ti_3_C_2_T*_x_* were widened and shifted toward lower angles, while the (104) peak disappeared (Figure 2d), suggesting that the Ti_3_AlC_2_ MAX phase was almost completely transformed into Ti_3_C_2_T*_x_* MXene [38].

Unlike mixing the MXene with sulfur or treating it using H_2_S, here, we facilitated the sulfidation by chemical vapor deposition (CVD), which uses a sulfur vapor to react with multilayer MXene particles. Sulfur demonstrates a relatively low boiling point and sublimation tendency. As a result, a reaction temperature exceeding the boiling point of elemental sulfur was set for the purpose of chemical vapor deposition. At a meticulously chosen temperature, sulfur gas interacts extensively with MXene, guaranteeing a consistent reaction. This deliberate regulation of temperature enhances the accuracy of the entire deposition process. In addition, unreacted sulfur is carried away by the gas flow to prevent excessive pollution. During the experiment, temperatures were controlled and vulcanized at 500 °C, 600 °C, 700 °C, and 800 °C, respectively.

The corresponding changes in the surface morphology and elemental composition of MX500 are shown in Figure 2a–c and Table S1. Morphologically, MX500 maintains its accordion-like structure, but the surface roughness increases. Significant signals were observed from the sulfur element in the EDS mapping image, which were uniformly distributed and better aligned with the distribution of titanium elements, suggesting a certain stoichiometric relationship between the sulfur and titanium elements. The XRD test was used to study changes in sample composition. Relevant data are shown in Figure 2d. Compared to Ti_3_C_2_T*_x_*, the XRD curve of MX500 shows not only the characteristic peak of Ti_3_C_2_T*_x_* but also of titanium disulfide (PDF#97-065-1203) and titanium dioxide (PDF#97-000-9853). This suggests that some of the MXene has been converted into titanium disulfide and titanium dioxide, which originate from oxygen-containing substances. In addition, the intensity of the (*00l*) peaks exceeded that of the characteristic peaks found on the standard card, which indicates a certain orientation of the growth of titanium disulfide.

TEM techniques were also employed to investigate the morphological and compositional changes in MX500. The HAADF image of MX500, along with its elemental distribution map (Figure S2a,b), shows a dominant presence of a sulfur element on the surface and edges of the sample, indicating that sulfidation mainly took place in these areas. Furthermore, the HR-TEM revealed that no crystal boundaries resembling those previously reported in the literature for single-layer sulfurization of MXene were observed [39], primarily due to the sulfurization method involving chemical vapor deposition, where elemental sulfur reacts with multilayered MXene in a gaseous state, guaranteeing consistent and thorough interaction and ensuring a thorough reaction.

As depicted in Figure 2e–g, the analysis of the lattice fringes through surface diffraction showed an average interplanar spacing of 0.19 nm. This aligns with the crystalline structure which was seen in the XRD patterns of TiS_2_, corresponding to the (003) crystal plane. These data provided further evidence that MXene had been converted into TiS_2_. Furthermore, the analysis using Fourier transformation analysis confirmed the presence of TiS_2_ on the surface of the sample. Based on the above results, it can be reasonably assumed that the formation mechanism of TiS_2_@TiO_2_@MXene is as follows: Ti_3_C_2_T*_x_* + S → Ti_3_C_2_T*_x_* + TiS_2_ + CS_2_ (g) [40]. Furthermore, partial TiS_2_ species can transform to TiO_2_, derived from the abundant -O/-OH terminations on the surface of MXene nanosheets [41].

With rising temperatures, the degree of sulfidation increases, and systematic changes occur in the morphological features (Figure S3a–d). This transformation occurs from the surface of MXene and progressively penetrates deeper into the interlayers, eventually converting MXene to TiS_2_ and TiO_2_. When the temperature of the CVD was heated to 600 °C, the accordion-like morphology began to deteriorate from the edges and surfaces, leading to the formation of hexagonal layers at the particle boundaries, which is indicative of TiS_2_. This further substantiates that the sulfidation reaction occurs at MXene’s surface and edges. As the temperature increased further, the original morphology was gradually destroyed. The accordion-like morphology was substituted with a mosaic-like appearance until the temperature hit 800 °C. Unsurprisingly, the elemental composition underwent regular changes, marked by a rise in sulfur levels with increasing temperatures. Interestingly, as the sulfurization degree rose, there wass a noticeable decline in the fluorine concentration. At 600 °C, the fluorine content neared zero, while at 700 °C and 800 °C, the fluorine elements remained undetectable. This indirectly verifies MXene’s sulfurization process.

As the temperature rose, noticeable changes in the samples’ composition were noted, which was thoroughly evidenced by the XRD data collected at different temperatures (Figure S4), and Figure S5a–d display the TEM images of MX600. Firstly, in comparison to samples subjected to a 500 °C treatment, higher temperatures induce a more comprehensive transformation of MXene. When heated to 600 °C, the (002) characteristic peak of MXene becomes almost invisible, leaving only the unique peaks of TiS_2_ and TiO_2_ distinctly noticeable. Additionally, as the temperature rises, the characteristic peaks associated with the anatase phase progressively attenuate, which is indicative of a phase transition into the Rutile phase. Notably, at 800 °C, the complete conversion of the Anatase phase into the Rutile phase is observed. We calculated the average crystallite sizes of MX500, MX600, MX700, and MX800 using the Scherrer Equation. The average crystallite sizes of MX500, MX600, MX700, and MX800 were 26.99 nm, 33.40 nm, 35.98 nm, and 31.42 nm. In the temperature range from 500 °C to 700 °C, the crystallite sizes increased. This was manifested in the X-ray diffraction (XRD) data by the disappearance of the characteristic peaks of MXenes and a narrower peak width, indicating an improvement in crystallinity. From 700 °C to 800 °C, there was a slight decrease in the average crystal size, although no significant change was observed in the XRD data, suggesting that there was no apparent phase transition. However, morphologically, the samples of MX700 appear flake-like, while the MX800 samples exhibit a columnar shape. This could be attributed to the fracture and restacking of TiS_2_ layers into columnar structures at high temperatures. This observation highlights the significant impact of temperature on both the samples’ structure and composition. XRD detected compositional changes, which offer a convincing reason for the significant lack of fluorine elements in the EDS data. This lack of fluorine aligns seamlessly with the ongoing sulfidation process of MXene.

### 2.2. Evolution of Structure and Surface

Raman spectroscopy was utilized for an in-depth examination of the sample’s surface properties to better understand and define its surface’s chemical composition. Ti_3_C_2_T*_x_* MXene exhibited vibrational patterns characterized by E_g_ (in-plane) and A_1g_ (out-of-plane) peaks, with the latter showing more uniqueness and intensity. Within the Raman spectrum (Figure 2h), the peak at 212 cm^−1^ corresponded to the A_1g_ (Ti, O, C) vibration mode, while the peak at 711 cm^−1^ matched the A_1g_ (C) mode. Additionally, a distinctive peak around 388 cm^−1^ is attributed to surface functional groups [42].

In contrast to MXene, the Raman spectrum of MX500 not only reveals a weakening or disappearance of the original MXene characteristic peaks but also manifests new features. Particularly, the peaks observed at 148 and 638 cm^−1^ are associated with titanium dioxide [43], while the peaks at 228 and 332 cm^−1^ suggest the presence of titanium disulfide [44]. Simultaneously, distinct carbon peaks at 1367 cm^−1^ and 1574 cm^−1^ are observed, stemming from the amorphous carbon that was formed during the transformation of Ti_3_C_2_T*_x_* into titanium disulfide [45]. This detailed Raman analysis provides insights into the structural evolution and chemical transformations occurring at the surface of the material, enhancing our overall grasp of its physicochemical characteristics. Under higher temperatures, a prominent peak at approximately 148 cm^−1^, indicative of the E_g_ mode, dominates the Raman spectrum (refer to Figure S6), along with characteristic peaks of A_1g_ (~508 cm^−1^) and E_g_ (~628 cm^−1^). The heights of these observed peaks align with the previously reported features, which is indicative of partial oxidation on the surface of the titanium disulfide at ~148 cm^−1^ [46].

**Figure 3:**
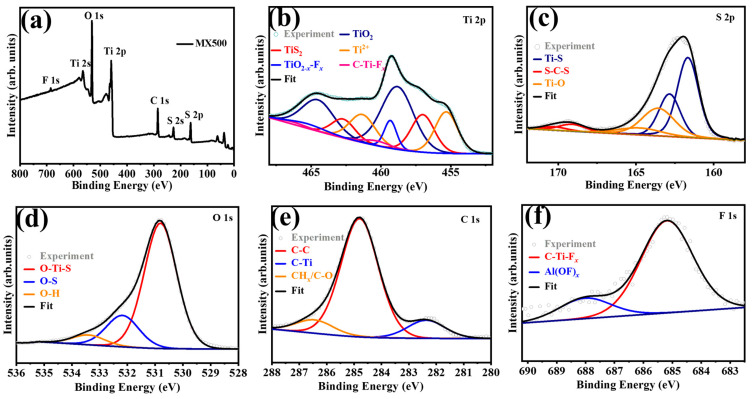
The XPS survey spectra of (**a**) MX500 and high-resolution (**b**) Ti 2p, (**c**) S 2p, (**d**) O 1s, (**e**) C 1s, and (**f**) F 1s spectra, the gray circles are experimental test data, and the black lines are fitted data.

The chemical states of MX500 were examined using XPS. The XPS survey spectra of MX500 are shown in Figure 3a, where the surface’s chemical composition corresponds with findings from SEM and TEM testing. In the detailed spectra analysis of Ti 2p (refer to Figure 3b), one can identify five unique pairs of peaks, located at 455.32/461.32 eV, 457/462.7 eV, 458.82/464.52 eV, 459.32/465.32 eV, and 460.3/466.3 eV and aligning with the Ti 2p_3/2_/2p_1/2_ orbitals of Ti^2+^, TiS_2_, TiO_2_, Ti-O_2-*x*_F*_x_*, and C-Ti-F*_x_* [47,48,49]. The detailed spectra analysis of S 2p (Figure 3c) reveals three pairs of peaks at 161.66/162.84 eV, 163.57/164.75 eV, and 169.11/170.29 eV, aligning with the S 2p_3/2_/2p_1/2_ orbitals of O-Ti-S, C-S-C, and S-O, respectively [49]. Figure 3d–f display the detailed spectra analysis of O 1s, C 1s, and F 1s. And the detailed XPS data for MX500 can be found in Table S2. The XPS analysis reveals that the material primarily consists of MXene, TiS_2_, and TiO_2_. Further exploration of the chemical composition of the samples that were exposed to sulfurization at 600 °C is detailed in Figure S7a–e and Table S3. The complete elimination of the Ti-C bond, the noticeable lack of the F 1s signal, and the comprehensive examination of the Ti 2p XPS spectra collectively signify the thorough transformation that is undergone by MXene.

### 2.3. Electrochemical Characterizations and the Lithium Ion Storage Mechanism

**Figure 4:**
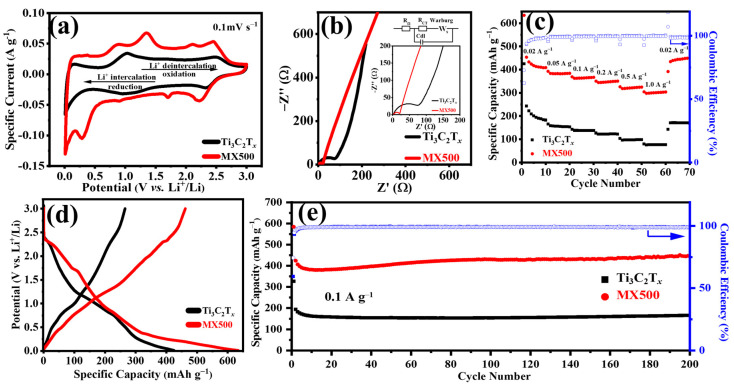
(**a**) CV curves of Ti_3_C_2_T*_x_* and MX500 at 0.1 mV s^−1^; (**b**) EIS plots of Ti_3_C_2_T*_x_* and MX500; (**c**) rate performance of Ti_3_C_2_T*_x_* and MX500; (**d**) first charge/discharge of Ti_3_C_2_T*_x_* and MX500 at 20 mA g^−1^; and (**e**) GCD curves of Ti_3_C_2_T*_x_* and MX500 at 0.1 A g^−1^; the blue points are the Coulomb efficiencies of different cycles.

To assess the Li^+^ storage capabilities of MX-n, 2032 coin-type cells were constructed, employing lithium foil as the counter electrode. The evaluation of their electrochemical reactions was conducted through cyclic voltammetry (CV), a multifaceted electroanalytical method that is renowned for its efficiency in studying electroactive species [50]. The remarkable usefulness of this method arises from its capacity to describe redox reactions quickly and thoroughly over a broad and adaptable spectrum of potentials. Figure 4a displays the CV curves of Ti_3_C_2_T*_x_* for MX500 at 0.1 mV s^−1^. The Ti_3_C_2_T*_x_* electrode shows normal redox peaks, and two pairs of oxidation/reduction peaks are observed near 2.53 V/2.45 V and 1.00 V/0.97 V [51]. Compared to the Ti_3_C_2_T*_x_* electrode, the MX500 electrode’s CV curve shows the following differences: Initially, the MX500 electrode exhibits an enhanced response current at similar potentials. This finding indicates an increased effectiveness in transferring lithium ions over a specific period, suggesting a greater capacity for lithium storage in the MX500 compared with the Ti_3_C_2_T*_x_* electrode, and the variation in the area that is covered by the CV curve further emphasizes this difference. Additionally, the CV curve revealed novel and unique oxidation reduction patterns, which are evident at potentials of 2.12 V/1.69 V and 0.86 V/0.29 V; the conversion between TiS_2_ and Li*_x_*TiS_2_ takes place at 2.12 V/1.69 V, while at 0.86 V, Li*_x_*TS_2_ converts to TiS_2_. Additionally, the chemical reaction occurring at 0.29 V involves the conversion of Li*_x_*TiS_2_ to Li_2_S and Ti [52]. Particularly, the peak in oxidation reduction at 1.3 V/0.89 V is associated with the insertion and extraction of lithium ions in titanium disulfide [53], and the peak at 2.05 V/1.7 V is linked to the insertion and extraction of lithium ions in titanium dioxide [54]. Moreover, changes were observed in the oxidation reduction peaks at 2.5 V/2.3 V and 1.0 V/0.9 V, marked by an increased peak intensity and a move to lower electrode potentials. This occurrence is linked to a series of oxidation reduction peaks in the titanium disulfide at 2.45 V/2.2 V. The merging and intersection of these peaks with MXene’s oxidation reduction peaks lead to a heightened peak intensity and a shift in peak locations [55]. Figure S8 illustrates the coefficient of variation graphs for the MX500 and MX600 samples. As the degree of sulfidation increases, the electrochemical oxidation reduction’s characteristic peaks of titanium disulfide undergo additional enhancement. Concurrently, MXene’s unique peaks experience additional weakening or total vanishing, causing a significant alteration in the peak potentials of the oxidation reduction towards those linked with titanium disulfide.

Figure 4b displays the distinct electrochemical impedance spectra (EIS) of MX500 and MXene, showcasing common patterns in lithium-ion batteries. Significantly, the semicircles that can be seen in areas of high- and medium-frequency regions outline the processes of charge transfer in lithium-ion electrode reactions. Within the low-frequency region, a diagonal line represents the diffusion process, clarifying the movement of ions diffusing in the solid phase [56]. The internal insets in Figure 4b illustrate the corresponding equivalent circuit and local curves derived from the EIS; a fitting of the electrode processes was performed, with the related numerical values being elaborated in Table S4. Compared to MXene, MX500 exhibited a minor rise in the solution resistance (R_s_), which is attributed to the lesser quantity of MXene and the heightened TiS_2_ and TiO_2_. However, there is a notable decrease in the interfacial transfer resistance (R_ct_), with R_ct_ dropping from 90.71 to 16.98 Ω. MX500 has a smaller Warburg impedance, suggesting faster lithium ion diffusion in the MX500 electrode, and MX500 has a larger CPE value, indicating a slightly poorer surface uniformity of MX500. This implies a hastened charge movement at the electrode interface for lithium ions in the MX500 electrode, showcasing its enhanced electrochemical efficiency.

### 2.4. Electrochemical Performance

It is essential for an electrode to exhibit remarkable reversibility in different rate conditions. The rate performances of both the Ti_3_C_2_T*_x_* and MX500 electrodes were systematically assessed across a spectrum of current densities, specifically, ones ranging from 0.02 A g^−1^ to 1.0 A g^−1^, as depicted in Figure 4c. MX500 exhibited an impressive specific capacity, starting at 435 mAh g^−1^ at 0.02 A g^−1^ and gradually decreasing as the current density increased to 300 mAh g^−1^ at 1.0 A g^−1^. When the current density reverted to 0.02 A g^−1^, the specific capacity surged back to 440 mAh g^−1^, achieving a remarkable coulombic efficiency of 98%. Significantly, MX500’s initial discharge-specific capacity increased from 424 to 634 mAh g^−1^ relative to Ti_3_C_2_T*_x_* (as depicted in Figure 4d), with its initial coulombic efficiency rising from 62.74% to 72.82%. This underscores the impressive performance that was demonstrated by MX500. MX500’s performance was further corroborated at various current densities. Although Ti_3_C_2_T*_x_* and MX500 both demonstrated remarkable stability, a significant disparity was noticeable at varying current densities. At a lower current density of 0.02 A g^−1^, the discharge-specific capacity of the MX500 electrode exceeded that of the Ti_3_C_2_T*_x_* electrode by approximately 2.5-fold. Additionally, MX500 exhibited a discharge-specific capacity that was approximately quadruple that of Ti_3_C_2_T*_x_* at a greater current density of 1.0 A g^−1^. The MX500 exhibited considerable cyclical stability. Even after 200 cycles at a current density of 0.1 A g^−1^, the MX500 electrode maintained a specific capacity of 449 mAh g^−1^, demonstrating an increasing pattern (Figure 4e). This observed phenomenon is due to increased spacing between layers in the samples, a result of lithium ions being intercalated and deintercalated [57]. This widened interlayer gap between multilayered MXenes remains stable, significantly boosting their capability to store lithium ions [58]. To delve deeper into the distinct abilities of MX500 at varying current densities, refer to Figure S9a–c. Following 200 cycles at varying current densities of 0.2, 0.5, and 1.0 A g^−1^, the specific capacities of 384, 280, and 294 mAh g^−1^, respectively, were shown. The unique composition and structure of MX500 can be credited for its improved capacity, cycling stability, and superior coulombic efficiency. The converted TiS_2_ and TiO_2_ significantly boosted this capacity, whereas the original MXene maintained its structural integrity, safeguarding it throughout the process of lithium-ion insertion/extraction. And Figure S9d displays the GCD curve of pure TiS_2_ at 0.1 A g^−1^, indicating inferior cycling stability compared to MX500.

Figure S10a illustrates a comparative analysis of the rate performance of samples obtained via CVD at different temperatures. These results show that with the temperature increases, there is a gradual increase in the discharge-specific capacity of the samples, which is linked to a higher content of TiS_2_ and TiO_2_. Nonetheless, the MX600 electrode demonstrates a poorer rate performance, and it even falls below that of MX500 at a current density of 1.0 A g^−1^. Unfortunately, despite the notable enhancement in the specific capacity observed in samples obtained at temperatures exceeding 500 °C, their cycling stability remains far from satisfactory. A rapid and noticeable trend of deterioration is observed during the charge/discharge (Figure S10b–d). The reason for this is the scarce or almost non-existent MXene in the samples, which hinders its ability to offer structural stability to TiS_2_ and TiO_2_ in the cycling phase, leading to the breakdown of electrode materials. The cyclic performance of the heterostructure materials reported in this paper is compared with those previously reported in Table S5; the materials reported in this paper show promising application prospects.

### 2.5. Electrochemical Kinetics

**Figure 5:**
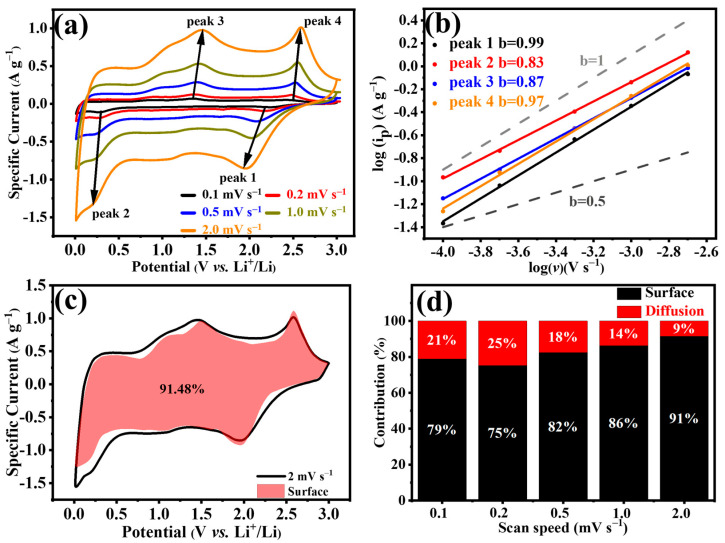
(**a**). CV curves of Ti_3_C_2_T*_x_* and MX500 at 0.1 mV s^−1^, 0.2 mV s^−1^, 0.5 mV s^−1^, 1.0 mV s^−1^, and 2.0 mV s^−1^; (**b**) b values of peak 1, peak 2, peak 3, and peak 4; (**c**) capacitive contribution and diffusion contribution at 2.0 mV s^−1^; (**d**) contribution ratio of capacitive capacities at different scan rates.

To investigate the transport process of lithium ions and the electrode kinetics, we performed CV measurements at various scanning rates from 0.1 to 2.0 mV s^−1^. Figure 5a displays the CV curves, where changes in the voltametric curve are noticeable as the scanning speed increases. Notably, there is an expansion in the peak width, and the peaks of oxidation reduction display a significant transition to higher potentials. Such fluctuations are intimately linked with the kinetics of lithium intercalation and deintercalation at the electrode–electrolyte interface, as well as the rate of lithium diffusion [59]. Equation (1) [60] outlines the correlation between the peak current (*i_p_*) and the scan rate (*v*).

(1) $$i=av^{b}$$

where “*a”* and “*b”* are adjustable variables, and the linear fitting values of “*b”* serve to determine if the dynamic process is inclined towards surface or diffusion control. Given that Equation (1) can be reformulated as Equation (2), the *b* value signifies the slope of the plot log(*i*) against log(*v*). Generally, when the slope of b equals 0.5, it indicates a diffusion-controlled process, which is characteristic of battery-type behavior. On the other hand, as the slope approaches 1, it indicates non-diffusion-controlled redox reactions predominantly occurring on the surface, emphasizing the capacitive effect or pseudocapacitance [61]. The b values for the four pairs of oxidation reduction peaks are 0.99, 0.83, 0.87, and 0.97, respectively (Figure 5b). The value of *b* lies between 0.5 and 1.0, leaning towards 1.0, indicating a higher dominance of the capacitive contribution.

(2) log(i)=blog⁡(v)+log⁡(a)

Moreover, as suggested by Conway and colleagues [62], one can quantitatively evaluate the current behavior of the surface control and diffusion control by calculating their respective proportions using the following formula:

(3) $$i=k_{1}v+k_{2}v^{\frac{1}{2}}$$

(4) $$i/v^{\frac{1}{2}}=k_{1}v^{\frac{1}{2}}+k_{2}$$

The current response (*i*) is bifurcated into two separate elements: surface-controlled processes (*k*_1_*ν*) and diffusion-controlled processes (*k*_2_*ν*^1/2^) at a constant potential (*ν*). By plotting *ν*^1/2^ against *i*/*ν*^1/2^, the values of *k*_1_ and *k*_2_ can be determined, enabling the computation of the pseudocapacitance’s impact. Figure 5c and Figure S11a–d illustrate the impact of the capacitance at varying scanning speeds. With the scan rate increases, it is clear that the impact of the diffusion-controlled contribution lessens, indicating that the primary contributor to the total capacity at elevated scan rates is the pseudocapacitive-controlled lithium storage. In particular, the distinct capacitive effect at 2.0 mV s^−1^ underscores that 91% of the CV area is linked to the capacitive effect. This dominance of a process with high capacity is due to the unique structure, which increases the interaction zone between the electrolyte and the active materials, thereby promoting pseudocapacitive actions. The percentage of the capacitive contribution to the overall capacity at scanning speeds of 0.1, 0.2, 0.5, and 1.0 mV·s^−1^ is, respectively, 79%, 75%, 82%, and 86% (Figure 5d). This suggests that MX500, derived from the conversion of MXene, exhibits not just an enhanced specific capacity and electrochemical cycling stability, but it also demonstrates pseudocapacitive traits akin to those of MXene. Additionally, we examined the kinetics of samples obtained at different temperatures. The diffusion and capacitive contributions of MX600, MX700, and MX800 at various scan rates are presented in Figure S12a–f, Figure S13a–f, and Figure S14a–f, respectively. Notably, each of these substances demonstrates remarkable pseudocapacitive properties, which are intimately linked to their MXene origin. Impressively, a significant capacitance is crucial in facilitating swift and reversible storage of ions [63].

## 3. Materials and Methods

### 3.1. Materials

The materials and reagents used in this study were as follows: Ti_3_AlC_2_ MAX powder (325 mesh, Jilin 11 Technology Co., Ltd. Jilin, China); hydrofluoric acid (49 wt%, Shanghai Aladdin Bio-Chem Technology Co., Ltd. Shanghai, China); sulfur powder (99.999%, Sinopharm Chemical Reagent Co., Ltd. Shanghai, China); polyvinylidene fluoride (Shenzhen Kejing Star Technology Co., Ltd. Shenzhen, China); and N-Methyl-2-Pyrrplidone (Shanghai Aladdin Bio-Chem Technology Co., Ltd. Shanghai, China).

### 3.2. Preparation of MX-n Heterostructures

#### 3.2.1. Preparation of MXenes

Ti_3_C_2_T*_x_* MXene was prepared by the wet-chemical etching method described previously. Briefly, 3 g of Ti_3_AlC_2_ MAX phase was slowly added to 60 mL of 10% HF solution. After etching for 18 h at 35 °C, the mixture was washed and centrifuged with deionized (DI) water until the pH value of the supernatant was ~6. Then, it was filtered and gathered the precipitate. For comparison, part of the precipitate was taken out and dried for 4 h in a vacuum oven at 110 °C.

#### 3.2.2. Preparation of MX-n Heterostructures

Firstly, 0.2 g of Ti_3_C_2_T*_x_* MXene powder was added to a 10 mL corundum crucible, and 2 g of sulfur powder was added to another one. Then, the two corundum crucibles were transferred to different locations of the tube furnace with double temperature areas. The sulfur was heated to 250 °C, and the Ti_3_C_2_T*_x_*powder was heated to aimed temperature, maintaining the temperature for 4 h, and then cooled down to room temperature at −5 °C/min. The samples were recorded as MX-n (*n* = 500, 600, 700, and 800).

#### 3.2.3. Preparation of Electrode and Cell Assembly

The working electrodes were prepared using MX-n materials; MX-n, super P, and PVDF were mixed in NMP, and the ratio of MX-n, super P, and PVDF was 8:1:1. Then, the mixture was coated onto 14 mm diameter copper films, followed by moving them to vacuum oven-dry them for 12 h at 80 °C. The CR2032 coin cell was assembled in an argon-filled glovebox. The 14 mm diameter lithium foil was used as the reference electrode. The PP separator was used, and 1M LiPF_6_ dissolved in an EC:DEC:DMC (volume ratio 1:1:1) solution was used as the battery electrolyte.

### 3.3. Material Characterization

X-ray diffraction (XRD, ADVANCE D8, Bruker, Billerica, MA, USA) with Cu Kα source and 2ϴ degree from 5° to 80° was used to identify the crystal structure and phase of the Ti_3_AlC_2_, Ti_3_C_2_T*_x_*, and MX-n materials. Scanning electron microscopy (SEM, 8230, Hitachi, Tokyo, Japan) with energy-dispersive spectroscopy (EDS) and transmission electron microscopy (TEM, Talos F200X, Thermo Fisher Scientific, Waltham, MA, USA) were used to identify samples’ morphology and elemental mapping. X-ray photoelectron spectroscopy (XPS, AXIS SUPRA+, SHIMADZU (CHINA) Co., Ltd. Kyoto, Japan) was performed in an XPS system with a monochromatic Al X-ray source, and the binding energy (BE) scales were assigned by adjusting the C 1s peak at 284.8 eV. Raman spectroscopy was applied to purify the chemical composition of the sample.

### 3.4. Electrochemical Measurements

Electrochemical measurements including galvanostatic charging/discharging (GCD), cyclic voltammetry (CV), and electrochemical impedance spectroscopy (EIS) were measured at 30 °C. The GCD charging/discharging tests were conducted on a LAND-CT2001C tester (Wuhan LAND Electronic Co., Ltd., Wuhan, China), and the CV and measurements were conducted using an electrochemical workstation (Bio-logic VMP3e, Seyssinet-Pariset, France); CV tests were carried out from 0.01 V to 3.0 V. The EIS measurements were performed in the 0.01–100 kHz frequency range.

## 4. Conclusions

To sum up, this research involves a detailed modification of Ti_3_C_2_T*_x_* MXene’s surface composition using the CVD technique. This method facilitates the transformation of multilayered MXene into layered TiS_2_ and TiO_2_, resulting in a range of advanced composite materials. The research delves into how MXene’s structure and composition transform under varying temperatures, uncovering the changes in the samples’ morphology and composition. The study of these materials revealed their enhanced specific capacities, with 435 mAh g^−1^ at 0.02 A g^−1^, and superior cycling stability for a capacity retention ratio of almost 100%, as evidenced by their electrochemical properties. An analysis was conducted on how the material’s structure affects its properties, along with an explanation of MXene’s function in composite heterostructure materials. Furthermore, the outcomes of the electrochemical kinetics study underscore the extraordinary pseudocapacitive capabilities and disclose the lithium-ion storage tendencies of these advanced substances. This research provides essential insights into MXene-based heterostructures and establishes an effective structural editing strategy via regulating the surface modifications of MXene for future applications. The implications of these findings are significant, establishing a foundation for advancements in materials engineering and electrochemical energy storage methods.

## Data Availability

Data are contained within the article.

## Supplementary Materials

The following supporting information can be downloaded at: https://www.mdpi.com/article/10.3390/molecules29081731/s1, Figure S1: (a) SEM image of Ti_3_C_2_T*_x_* and (b) its corresponding elemental distribution mapping (Ti: yellow, C: red, O: green, F: blue); Figure S2: (a) HAADF-TEM image of MX500 and (b) its corresponding elemental distribution mapping (Ti: green, C: light blue, O: red, F: purple); Figure S3: SEM images of (a) MX500, (b) MX600, (c) MX700, and (d) MX800; Figure S4: XRD curves of MX500, MX600, MX700, and MX800; Figure S5: (a) TEM image of MX600, (b) HRTEM image of MX600, and (c) the image of the selected area in (b), and d. FFT pattern of (b); Figure S6: Raman spectra of MX500, MX600, MX700, and MX800; Figure S7: XPS survey spectra of (a) MX600, and high-resolution (b) Ti 2p, (c) S 2p, (d) C 1s, and (e) O 1s spectra.; Figure S8: CV curves of MX500 and MX600; Figure S9: GCD curves of Ti_3_C_2_T*_x_* and MX500 at (a) 0.2 A g^-1^, (b) 0.5 A g^-1^, and (c) 1.0 A g^-1^, (d) GCD curves of pure TiS_2_; Figure S10: (a) Rate capabilities of MX-n, Cycle performances of MX-n at the current density of (b) 0.1 A g^-1^, (c) 0.2 A g^-1^, and (d) 0.5 A g^-1^; Figure S11: Capacitive contribution and diffusion contribution for MX500 at (a) 0.1 mV s^-1^, (b) 0.2 mV s^-1^, (c) 0.5 mV s^-1^, and (d) 1.0 mV s^-1^; Figure S12: Capacitive contribution and diffusion contribution for MX600 at (a) 0.1 mV s^-1^, (b) 0.2 mV s^-1^, (c) 0.5 mV s^-1^, (d) 1.0 mV s^-1^, (e) 2.0 mV s^-1^, (f) contribution ratio of capacitive capacities at different scan rates; Figure S13: Capacitive contribution and diffusion contribution for MX700 at (a) 0.1 mV s^-1^, (b) 0.2 mV s^-1^, (c) 0.5 mV s^-1^, (d) 1.0 mV s^-1^, (e) 2.0 mV s^-1^, (f) contribution ratio of capacitive capacities at different scan rates; Figure S14: Capacitive contribution and diffusion contribution for MX800 at (a) 0.1 mV s^-1^, (b) 0.2 mV s^-1^, (c) 0.5 mV s^-1^, (d) 1.0 mV s^-1^, (e) 2.0 mV s^-1^, (f) contribution ratio of capacitive capacities at different scan rates; Table S1: Relative atomic composition tables of different samples (vs. Ti atom); Table S2: XPS analysis of MX500; Table S3: XPS analysis of MX600; Table S4: EIS fitting data; Table S5: Comparison with other materials as LIBs anodes. References [41,46,48,49,64,65,66,67,68,69,70,71,72,73,74,75,76,77,78] are cited in the supplementary materials.
