# Supplementary material for: Engineering Ti3C2-MXene Surface Composition for Excellent Li+ Storage Performance
Source: Molecules. 2024 Apr 11;29(8):1731. doi: 10.3390/molecules29081731 (PMC11052082; doi:10.3390/molecules29081731)
Supplement: Supplementary file 1 [file molecules-29-01731-s001.zip › molecules-2945093-supplementary.pdf]

Supplementary Materials

Engineering Ti_3C_2 MXene Surface Composition with Excellent Li^+ Storage Performance

Minghua Chen[1,2], Qi Fan[2,3], Ping Yu[4], Ke Chen[2,5], Peng Li[2,5], Kun Liang[2,3,5*]

[1] School of Materials Science and Chemical Engineering, Ningbo University, Ningbo, Zhejiang 315211, China

[2] Zhejiang Key Laboratory of Data-Driven High-Safety Energy Materials and Applications, Ningbo Key Laboratory of Special Energy Materials and Chemistry, Ningbo Institute of Materials Technology and Engineering, Chinese Academy of Sciences, Ningbo 315201, China.

[3] University of Chinese Academy of Sciences, 19 A Yuquan Rd, Shijingshan District, Beijing 100049, China.

[4] School of Electronic and Information Engineering, Ningbo University of Technology, 315211

[5] Qianwan Institute of CNITECH, Ningbo 315336, China.

*Corresponding Authors: E-mail: kliang@nimte.ac.cn

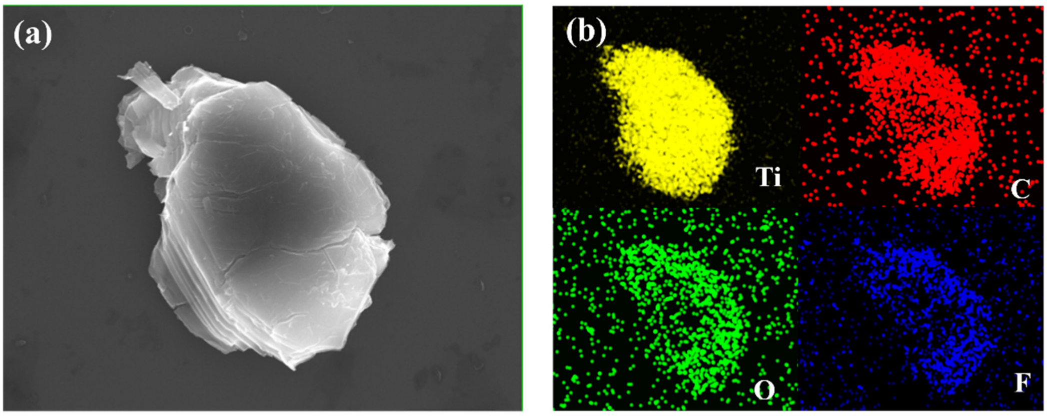

Figure S1. (a) SEM image of $Ti_3C_2T_x$ and (b) its corresponding elemental distribution mapping (Ti: yellow, C: red, O: green, F: blue).

Figure S1(a) shows the scanning electron microscope (SEM) image of MXene prepared through hydrofluoric acid etching, and **Figure S1(b)** is its corresponding elemental distribution map. The disappearance of aluminum and the uniform distribution of constituent elements both indicate the successful etching process.

Table S1 Relative atomic composition tables of different samples (vs. Ti atom)

Sample	Ti	C	Al	F	O	S
**$Ti_3C_2T_x$**	3	4.45	0.04	1.09	1.47	0
**MX500**	3	3.16	0.03	0.10	3.92	2.40
**MX600**	3	4.25	0.03	0.06	3.72	3.50
**MX700**	3	1.82	0	0	0.73	4.07
**MX800**	3	3.33	0	0	1.48	4.85

Table S1 delineates the relative atomic compositions of diverse samples, derived from EDS data. The findings illuminate a proportional augmentation in sulfur content concomitant with ascending temperatures. Notably, at temperatures exceeding 600 °C, fluorine (F) elements undergo total elimination, signaling the thorough sulfurization of the MXene structure.

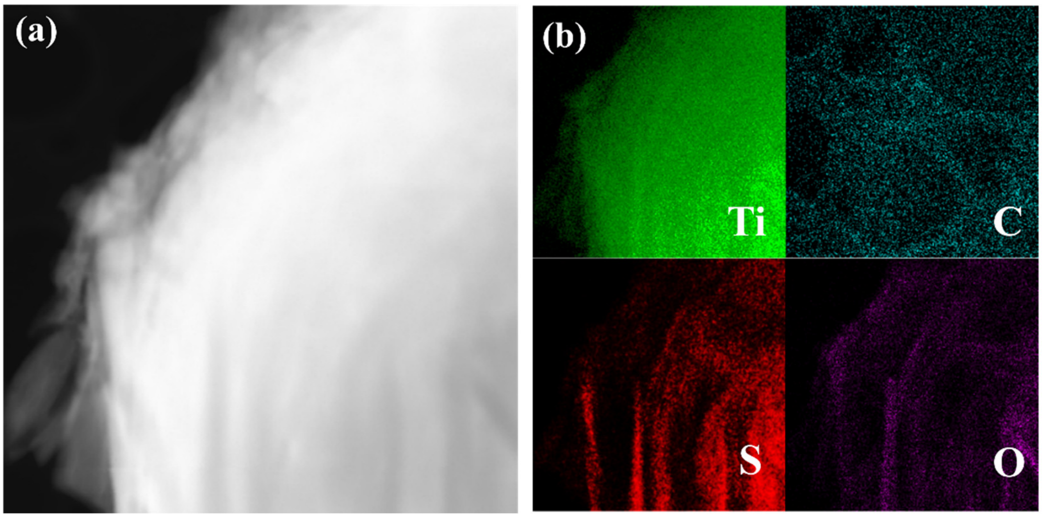

Figure S2. (a) HAADF-TEM image of MX500 and (b) its corresponding elemental distribution mapping (Ti: green, C: light blue, O: red, F: purple).

Figure S2(a) depicts an image of MX500 taken under dark-field conditions, and **Figure S2(b)** illustrates the distribution of constituent elements. The results indicate a more concentrated distribution of sulfur elements at the material's edges and surfaces, consistent with the expected primary reaction sites.

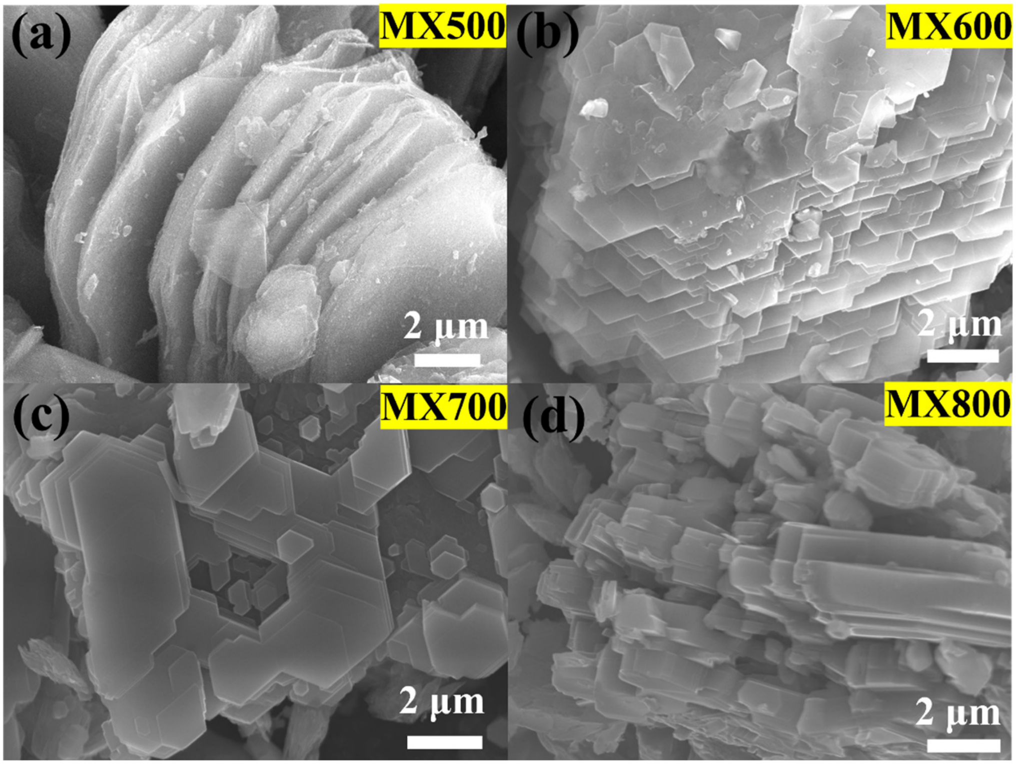

Figure S3 SEM images of (a) MX500, (b) MX600, (c) MX700, and (d) MX800.

The SEM images depicted in **Figures S3(a)-3(d)** illustrate the morphological transformations of samples subjected to sulfuration treatments at temperatures of 500 °C, 600 °C, 700 °C, and 800 °C. As the temperature elevates, discernible alterations manifest on the specimens' surface and periphery. The initially observed accordion-like morphology of MXene undergoes a gradual evolution, transitioning into

stratified layers characterized by a hexagonal structure of sulfurized titanium. Notably, at the pinnacle temperature of 800 °C, the samples exhibit a distinctive rectangular prismatic mosaic morphology, intricately linked to the extent of sulfuration.

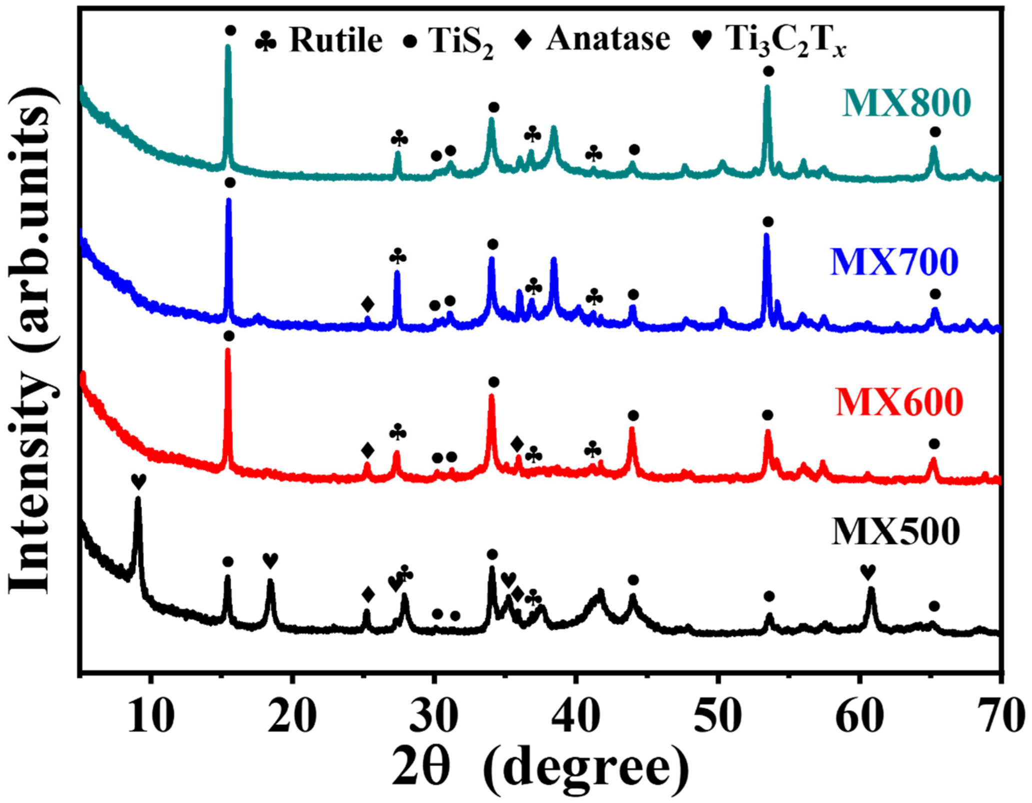

Figure S4 XRD curves of MX500, MX600, MX700, and MX800

Figure S4 presents the X-ray diffraction (XRD) patterns of samples subjected to sulfuration treatments at varying temperatures. The findings suggest that, with the temperature elevation to 600°C or beyond, the distinctive diffraction patterns associated with MXene become imperceptible in the XRD data, aligning consistently with both morphological observations and energy-dispersive X-ray spectroscopy (EDS) results. Notably, at 800°C, titanium dioxide undergoes a comprehensive transition from the Anatase phase to the Rutile phase.

Table S2 XPS analysis of MX500

Region	BE (eV)	FWHM (eV)	Assigned to	Ref.
**Ti 2p3/2(2p 1/2)**	455.32(461.32)	1.90(2.16)	Ti^{2+}	[1]
	457.00(462.70)	1.96(1.96)	TiS_2	[2]
	458.82(464.52)	2.71(2.71)	TiO_2	[1]
	459.32(465.32)	1.04(3.16)	$Ti\text{-}O_{2\text{-}x}F_x$	[1]
	460.3(466.30)	2.29(2.39)	$C\text{-}Ti\text{-}F_x$	[1]
**S 2p 3/2(2p 1/2)**	161.66(162.84)	1.61(1.61)	Ti-S	[2]
	169.11(170.29)	1.98(1.98)	S-O	[2]

	163.57(164.75)	2.45(3.26)	C-S-C	[2, 3]
**O 1s**	530.81	1.38	O-Ti-S	[1]
	532.18	1.38	O-S	[1]
	533.40	1.38	O-H	[4]
**C 1s**	282.37	1.60	Ti-C	[1]
	284.80	1.60	C-C	[1]
	286.45	1.60	C-O	[1]
**F 1s**	685.18	2.12	C-Ti-F_x	[1]
	688.02	2.12	Al(OF)$_x$	[1]

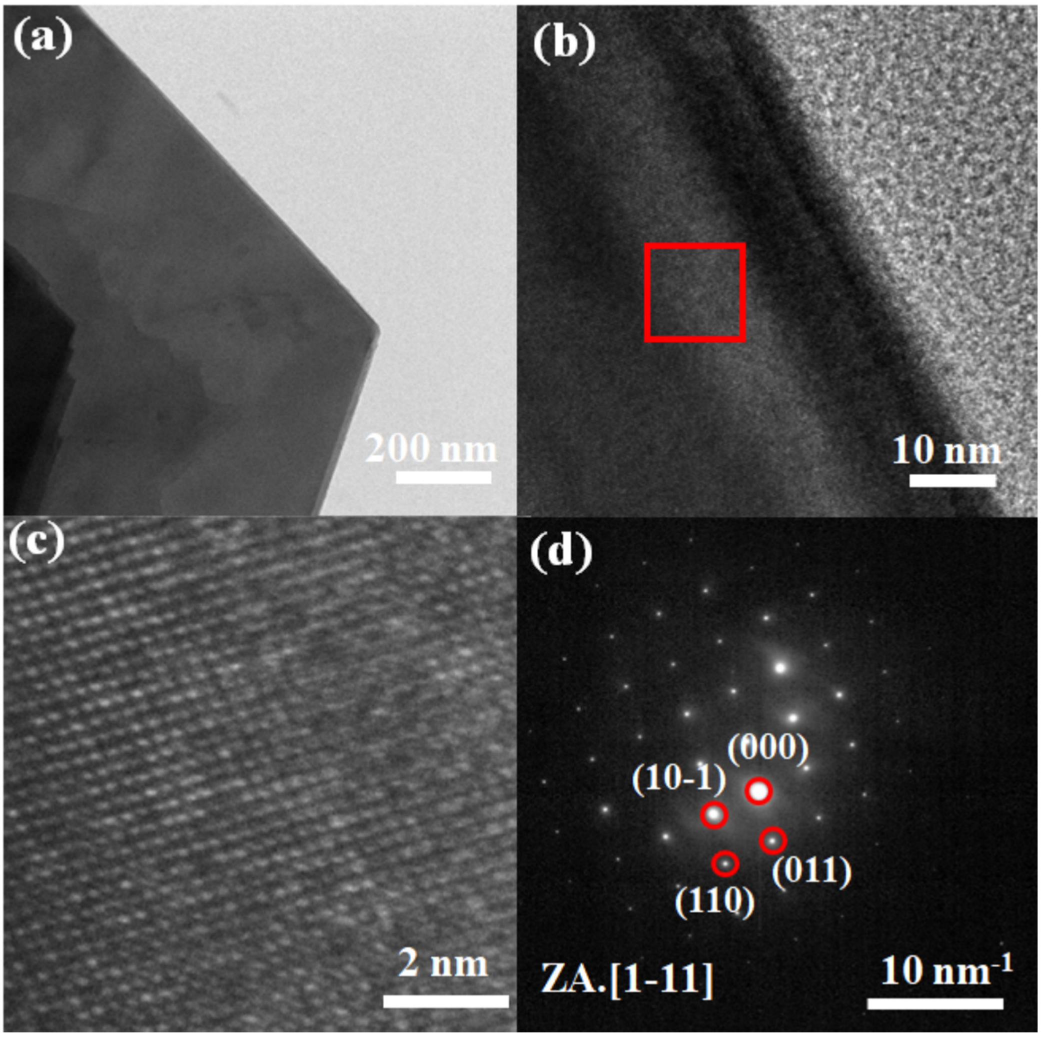

Figure S5. (a) TEM image of MX600, (b) HRTEM image of MX600, and (c) the image of the selected area in (b), and d. FFT pattern of (b).

Figure S5(a) presents the TEM image of MX600, and **Figure S5(b)** displays the HRTEM image, while

Figure S5(d) shows diffraction spots in the FFT pattern. Morphologically, MXene has completely transformed into titanium disulfide with a hexagonal structure, as evidenced by diffraction spots corresponding to titanium disulfide composition.

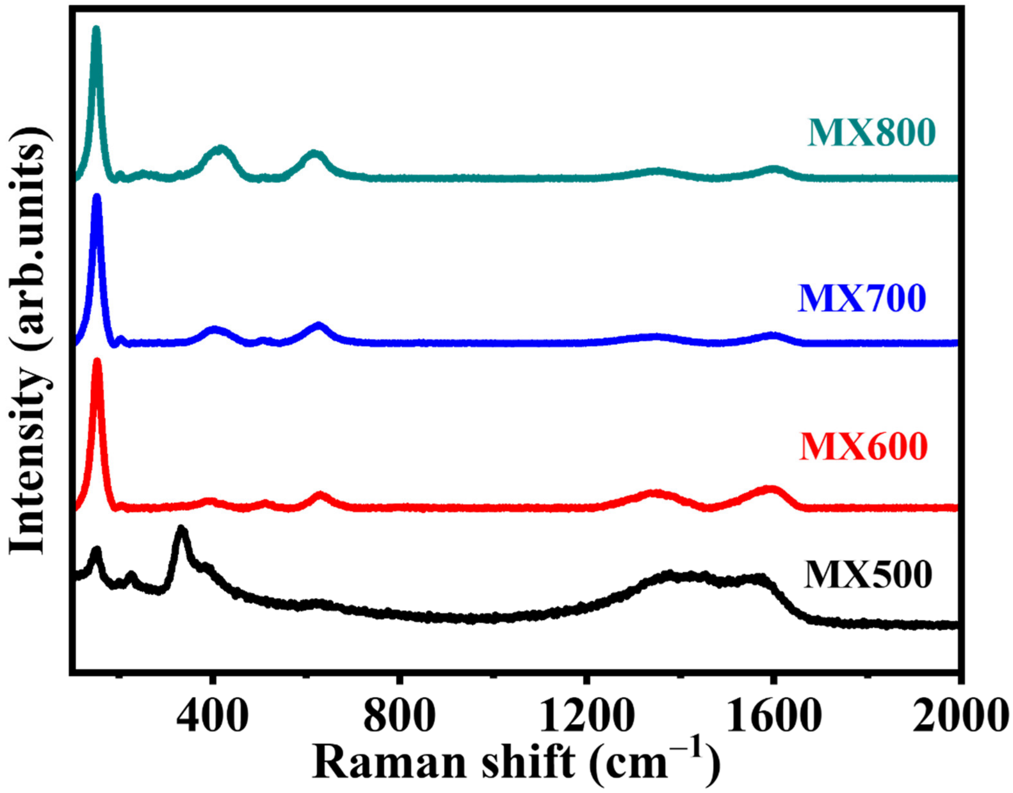

Figure S6 Raman spectra of MX500, MX600, MX700, and MX800.

Figure S6 displays the Raman spectra of samples treated with sulfurization at different temperatures. Apart from MX500, the remaining samples exhibit similar structural features. These results are consistent with previously reported partially oxidized titanium disulfide [5].

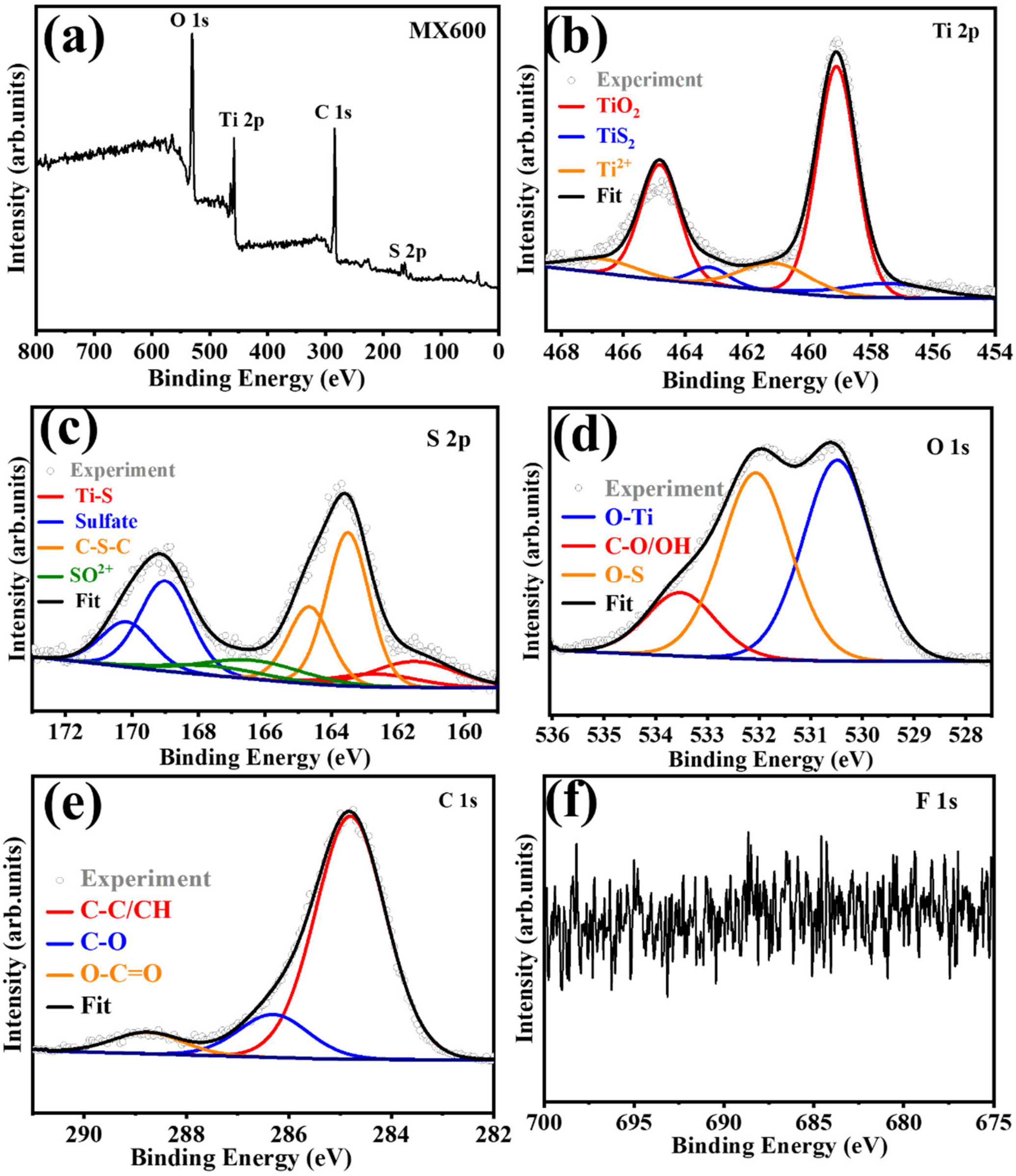

Figure S7. XPS survey spectra of (a) MX600, and high-resolution (b) Ti 2p, (c) S 2p, (d) C 1s, and (e) O 1s spectra.

Table S3 XPS analysis of MX600

Region	BE (eV)	FWHM (eV)	Assigned to	Ref.
**Ti 2p $_{3/2}$(2p $_{1/2}$)**	461.09(466.69)	2.66(2.66)	Ti^{2+}	[1]
	457.49(463.19)	3.56(1.42)	TiS_2	[2]
	459.12(464.82)	1.49(1.49)	TiO_2	[1]
**S 2p $_{3/2}$(2p $_{1/2}$)**	161.50(162.66)	2.96(2.96)	Ti-S	[2]
	163.50(164.66)	1.50(1.50)	C-S-C	[3]
	166.39(167.55)	3.60(3.60)	SO_3^{2+}	[2]
	169.00(170.16)	1.81(1.81)	sulfate	[3]
**O 1s**	530.48	1.58	O-Ti	[1]
	532.06	1.58	O-S	[2]
	533.52	1.58	O-H	[1]
**C 1s**	284.80	1.61	C-C	[1]
	286.30	1.61	C-O	[1]
	288.76	1.61	O-C=O	[3]

Figures S7(a)-S7(f) and **Table S3** present the X-ray photoelectron spectroscopy data for MX600. The signal for fluorine elements has been notably absent, and the presence of Ti-C bonds is not observed. This indicates a complete transformation of MXene, with titanium elements predominantly existing in the forms of titanium oxide and titanium disulfide.

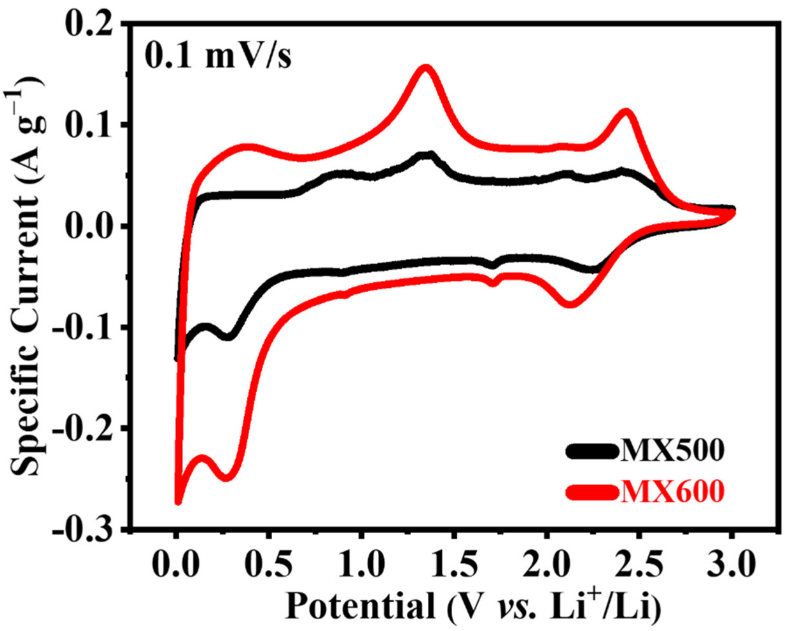

Figure S8. CV curves of MX500 and MX600.

Figure S8 presents a comparison of the CV curves between MX500 and MX600 samples. In comparison to MX500, the oxidation-reduction peak values of the MX600 sample shift towards lower potentials, indicating a further deepening of the sulfuration degree.

Table S4 EIS fitting data

Example	R_s (Ω)	R_{ct} (Ω)	W_d (Ω cm^2)	CPE (F cm^{-2})
**$Ti_3C_2T_x$**	2.00	90.71	9.38	0.80
**MX500**	3.30	16.98	1.44	0.83

Table S4 provides detailed data obtained through fitting EIS with an equivalent circuit. Compared to $Ti_3C_2T_x$, MX500 exhibits a smaller interfacial charge transfer resistance, indicating better charge transfer performance; a smaller Warburg impedance, suggesting faster lithium-ion diffusion in the MX500 electrode; and a larger CPE value, indicating slightly poorer surface uniformity of MX500.

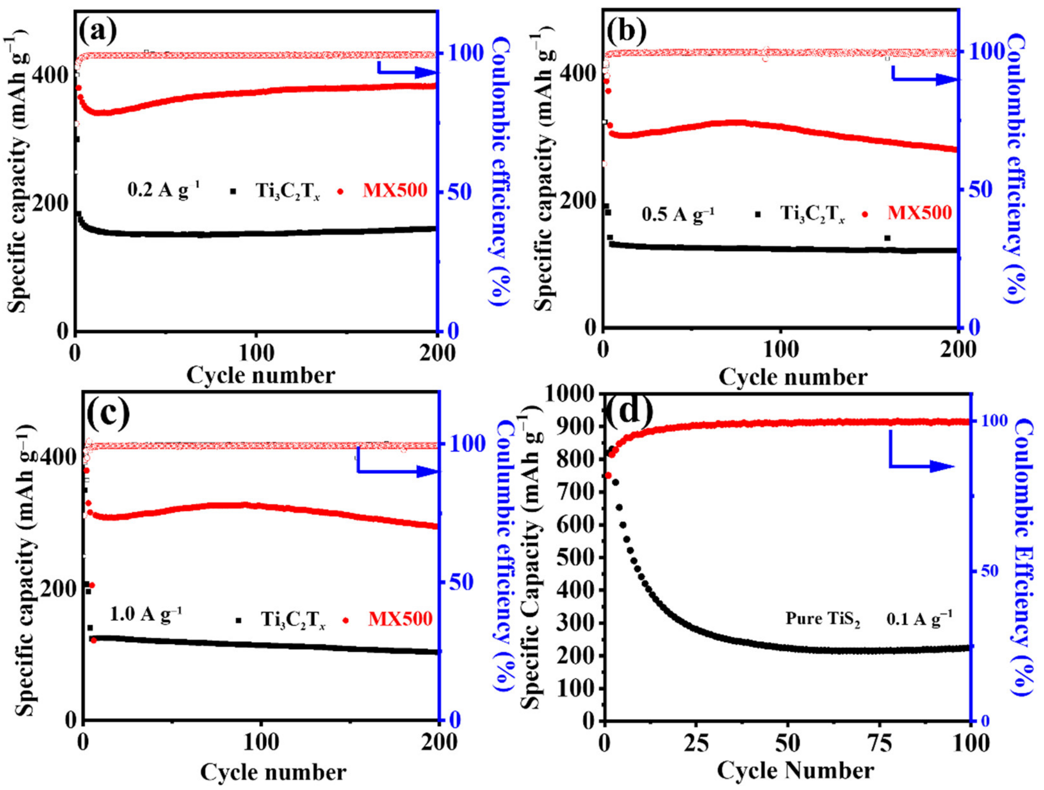

Figure S9 GCD curves of $Ti_3C_2T_x$ and MX500 at (a) 0.2 A g^{-1}, (b) 0.5 A g^{-1}, and (c) 1.0 A g^{-1}, (d) GCD curves of pure TiS_2.

Figures S9(a)-(c) depict the discharge data of $Ti_3C_2T_x$ and MX500 at discharge current densities of 0.2 A g^{-1}, 0.5 Ag^{-1}, and 1.0 A g^{-1}. Despite both exhibiting good cycling stability, MX500 demonstrates approximately twice the specific capacity compared to $Ti_3C_2T_x$. This suggests that MX500 exhibits superior lithium-ion storage capability. Figure S9(d) illustrates the discharge data of pure TiS_2 at a current density of 0.1 A g^{-1}, indicating inferior cycling stability compared to MX500.

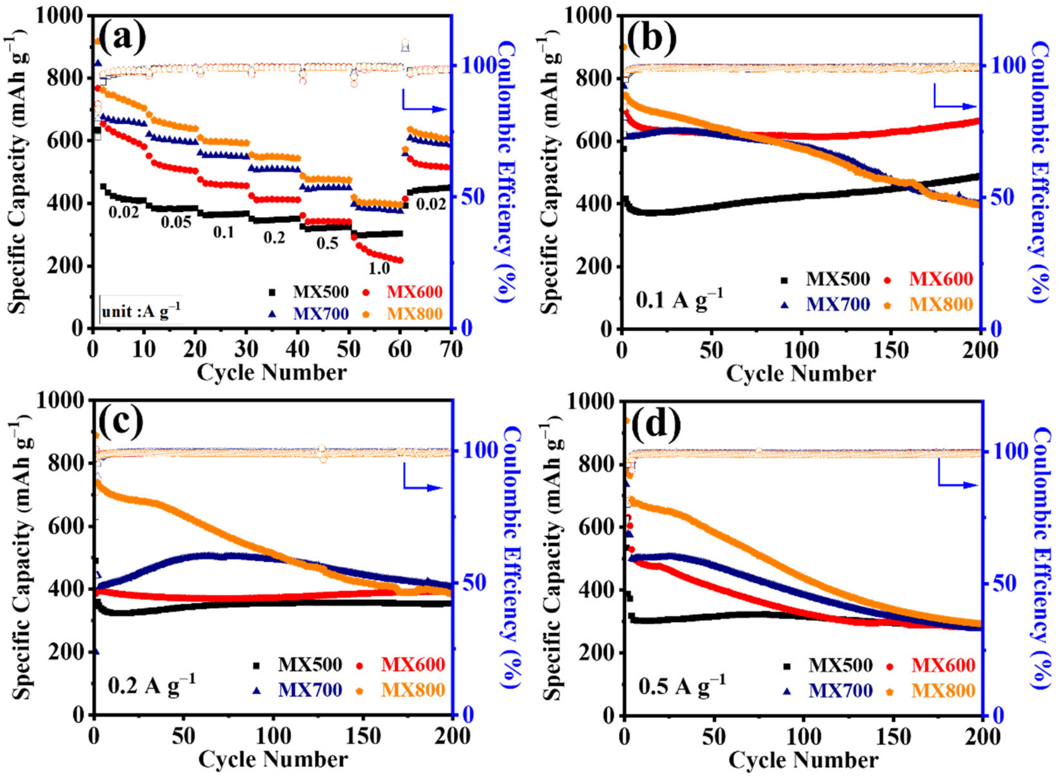

Figure S10. (a) Rate capabilities of MX-n, Cycle performances of MX-n at the current density of (b) 0.1 A g^{-1}, (c) 0.2 A g^{-1}, and (d) 0.5 A g^{-1}.

Figure S10(a) delineates the rate performance curves of samples subjected to varied sulfuration treatments. Except for MX600, which manifests relatively diminished rate performance at a current density of 1.0 A g^{-1}, the overall rate performance of the remaining samples is commendable. Furthermore, an observable correlation exists between the increasing sulfuration degree and the overall rising capacity of the samples. In **Figures S10(b)-S10(d),** discharge capacity performance curves are presented at constant current densities of 0.1 A g^{-1}, 0.2 A g^{-1}, and 0.5 A g^{-1}. At current densities of 0.1 A g^{-1} and 0.2 A g^{-1}, both MX500 and MX600 exhibit stable cycling behavior, while MX700 and MX800 display comparatively inferior performance. Nevertheless, with an escalation in discharge current density to 0.5 A g^{-1}, the cycling performance of MX600 experiences a corresponding decline.

Table S5 Comparison with other materials as LIBs anodes

Electrode materials	Current density	Specific capacity (mAh g^{-1})	Ref.
**MX500**	0.1 A g^{-1}	449	This work
**MX600**	0.1 A g^{-1}	663	This work
**MX700**	0.1 A g^{-1}	403	This work
**MX800**	0.1 A g^{-1}	403	This work
**Pure TiS_2**	0.1 A g^{-1}	226	This work
**$Ti_3C_2T_x$**	1 C	123.6	[6]
**Ti_3C_2/TiO_2/rGO**	1 A g^{-1}	176	[7]
**TiO_2/Ti_3C_2**	0.2 A g^{-1}	267	[8]
**Fe_3O_4@Ti_3C_2**	1 C	382.9	[9]
**MA-Ti_3C_2**	0.1 A g^{-1}	230	[10]
**Ti_3C_2/NiCo-MOF**	1 A g^{-1}	256	[11]
**Ti_3C_2/GO**	0.1 A g^{-1}	493	[12]
**CNT@Ti_3C_2**	10 A g^{-1}	175	[13]
**Ti_3C_2@CNF**	0.04 A g^{-1}	306.5	[14]
**TiO_2**	0.02 A g^{-1}	151.33	[15]
**TiS_2@Ti_2CS_2**	Theoretical	566	[16]
**TiS_2@NSC**	0.2 C	110	[17]
**Graphite**	Theoretical	372	[18]
**Nb_2C**	1 C	170	[19]

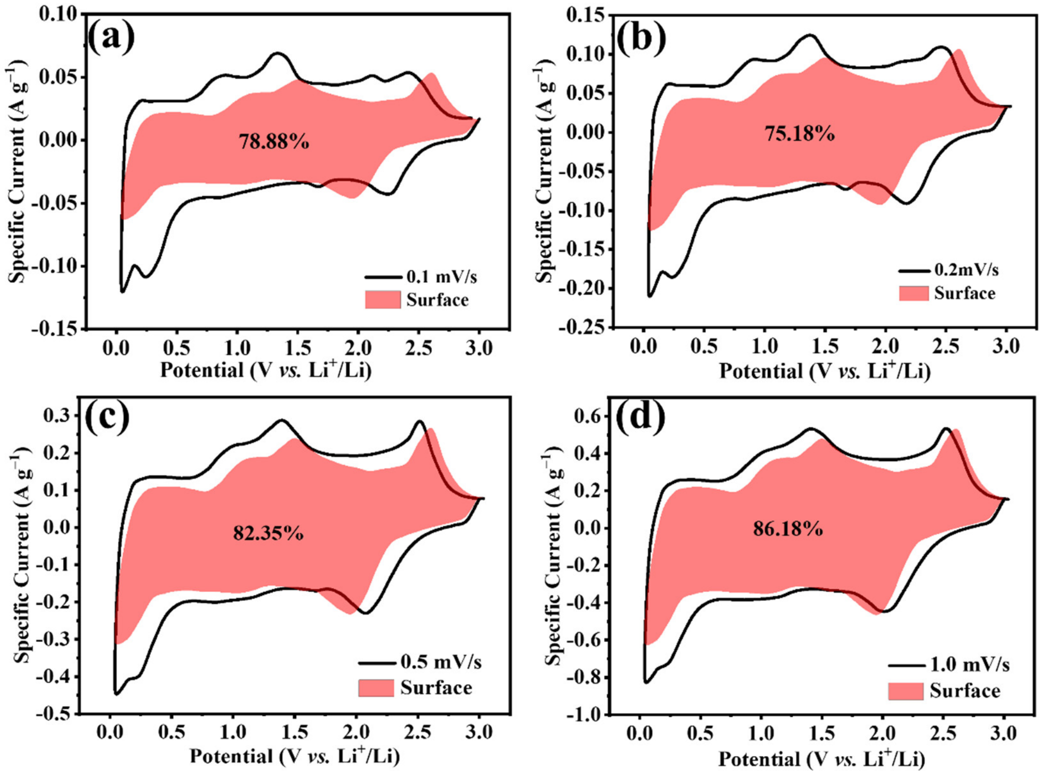

Figure S11 Capacitive contribution and diffusion contribution for MX500 at (a) 0.1 mV s^{-1}, (b) 0.2 mV s^{-1}, (c) 0.5 mV s^{-1}, and (d) 1.0 mV s^{-1}.

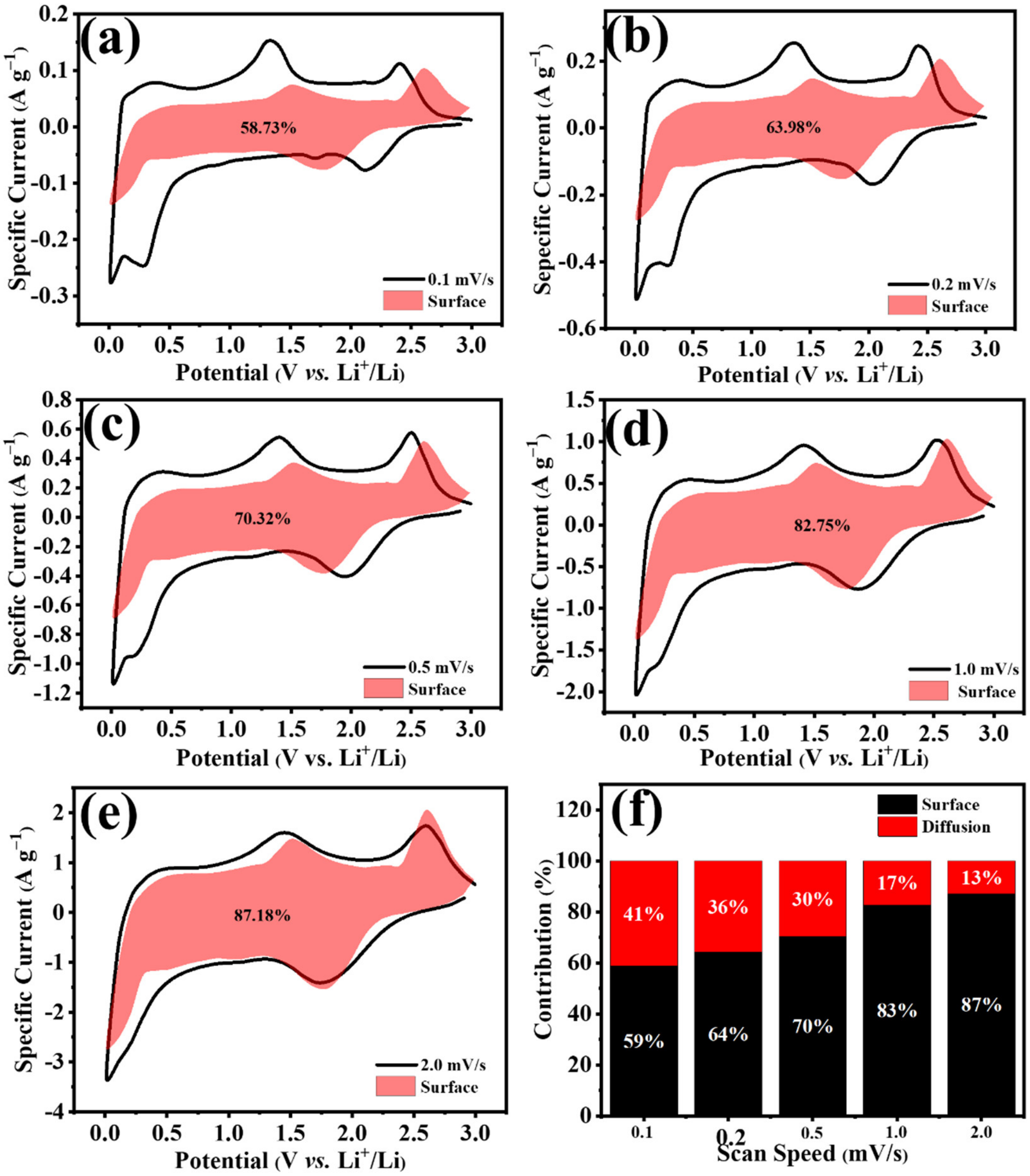

Figure S12. Capacitive contribution and diffusion contribution for MX600 at (a) 0.1 mV s^{-1}, (b) 0.2 mV s^{-1}, (c) 0.5 mV s^{-1}, (d) 1.0 mV s^{-1}, (e) 2.0 mV s^{-1}, (f) contribution ratio of capacitive capacities at different scan rates.

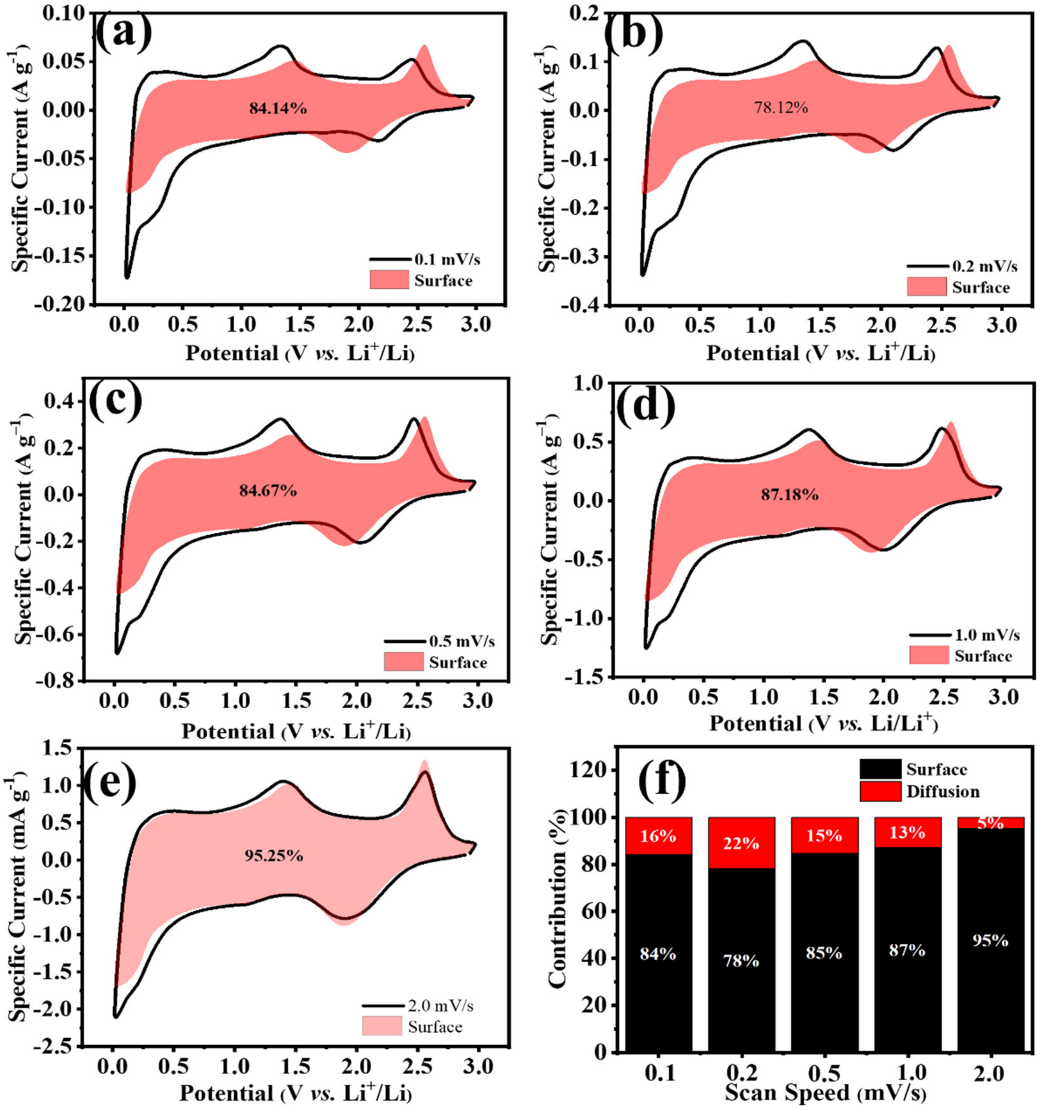

Figure S13. Capacitive contribution and diffusion contribution for MX700 at (a) 0.1 mV s^{-1}, (b) 0.2 mV s^{-1}, (c) 0.5 mV s^{-1}, (d) 1.0 mV s^{-1}, (e) 2.0 mV s^{-1}, (f) contribution ratio of capacitive capacities at different scan rates.

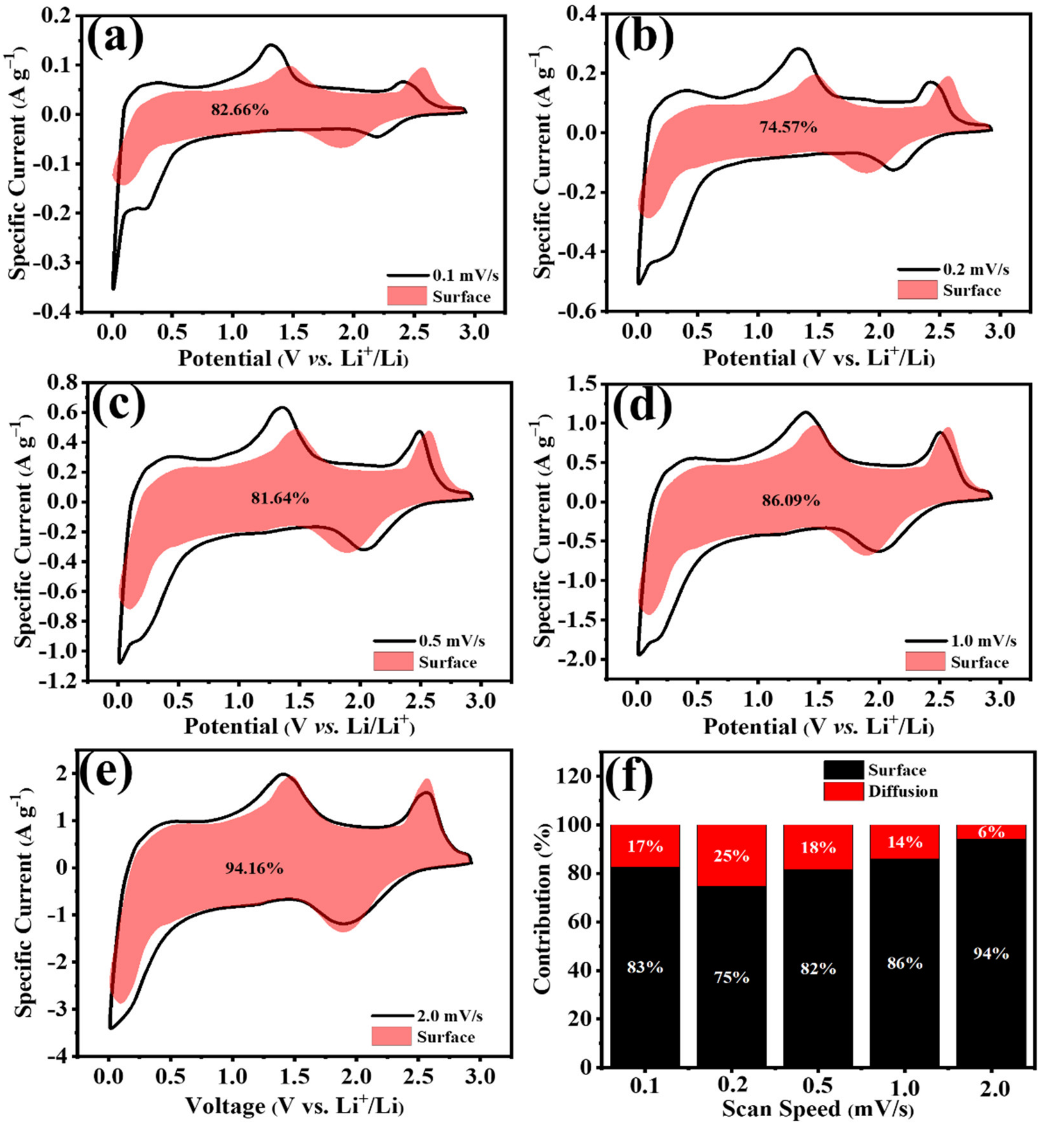

Figure S14. Capacitive contribution and diffusion contribution for MX800 at (a) 0.1 mV s^{-1}, (b) 0.2 mV s^{-1}, (c) 0.5 mV s^{-1}, (d) 1.0 mV s^{-1}, (e) 2.0 mV s^{-1}, (f) contribution ratio of capacitive capacities at different scan rates.

Figures S12(a)-12(f), S13(a)-13(f), and **S14(a)-14(f)** delineate the ratios of pseudocapacitive contributions to diffusion contributions for MX600, MX700, and MX800 samples across various scan rates. Notably, all MX-n samples manifest conspicuous pseudocapacitive contributions, affirming that specimens undergoing MXene sulfuration treatment retain distinctive pseudocapacitive characteristics.

Reference

1. Ghidiu, M.; Halim, J.; Kota, S.; Bish, D.; Gogotsi, Y.; Barsoum, M. W. Ion-exchange and cation solvation reactions in Ti_3C_2 MXene. *Chem. Mat.* **2016**, *28,* 3507-3514.

2. Huckaba, A. J.; Gharibzadeh, S.; Ralaiarisoa, M.; Roldán-Carmona, C.; Mohammadian, N.; Grancini, G.; Lee, Y.; Amsalem, P.; Plichta, E. J.; Koch, N.; Moshaii, A.; Nazeeruddin, M. K. Low-cost TiS_2 as hole-transport material for perovskite solar cells. *Small Methods* **2017**, *1,* 1700250.

3. Wagner, C. D.; Naumkin, A. V.; Kraut-Vass, A.; Allison, J. W.; Powell, C. J.; Rumble Jr, J. R. NIST standard reference database 20. **2003**,

4. Yamamoto, S.; Bluhm, H.; Andersson, K.; Ketteler, G.; Ogasawara, H.; Salmeron, M.; Nilsson, A. In situx-ray photoelectron spectroscopy studies of water on metals and oxides at ambient conditions. *J. Phys. Condens. Matter.* **2008**, *20,*

5. Sharma, S.; Singh, S.; Singh, R. C.; Sharma, S. Structural transformation and room temperature ammonia sensing properties of TiS_2 nanostructures. *SN Appl. Sci.* **2020**, *2,* 887.

6. Sun, D.; Wang, M.; Li, Z.; Fan, G.; Fan, L.-Z.; Zhou, A. Two-dimensional Ti_3C_2 as anode material for Li-ion batteries. *Electrochem. Commun.* **2014**, *47,* 80-83.

7. Li, Z.; Chen, G.; Deng, J.; Li, D.; Yan, T.; An, Z.; Shi, L.; Zhang, D. Creating sandwich-like Ti_3C_2/TiO_2/rGO as anode materials with high energy and power density for Li-ion hybrid capacitors. *ACS Sustain. Chem. Eng.* **2019**, *7,* 15394-15403.

8. Yang, C.; Liu, Y.; Sun, X.; Zhang, Y.; Hou, L.; Zhang, Q.; Yuan, C. In-situ construction of hierarchical accordion-like TiO_2/Ti_3C_2 nanohybrid as anode material for lithium and sodium ion batteries. *Electrochim. Acta* **2018**, *271,* 165-172.

9. Kong, F.; He, X.; Liu, Q.; Qi, X.; Sun, D.; Zheng, Y.; Wang, R.; Bai, Y. Further surface modification by carbon coating for in-situ growth of Fe_3O_4 nanoparticles on MXene Ti_3C_2 multilayers for advanced Li-ion storage. *Electrochim. Acta* **2018**, *289,* 228-237.

10. Liu, M.-C.; Zhang, B.-M.; Zhang, Y.-S.; Hu, Y.-X. Diacid molecules welding achieved self-adaption layered structure Ti_3C_2 MXene toward fast and stable lithium-ion storage. *ACS Sustain. Chem. Eng.* **2021**, *9,* 12930-12939.

11. Liu, Y.; He, Y.; Vargun, E.; Plachy, T.; Saha, P.; Cheng, Q. 3D porous Ti_3C_2 MXene/NiCo-MOF composites for enhanced lithium storage. *Nanomaterials* **2020**, *10,* 695.

12. Wang, Y.-Q.; Zhang, D.-T.; Zhao, B.; Chen, H.; Chang, C.-G.; Liu, M.-C. Ti_3C_2/graphene oxide layered nanocomposites for enhanced lithium-ion storage. *ACS Appl. Nano Mater.* **2023**, *6,* 3572-3579.

13. Zheng, W.; Zhang, P.; Chen, J.; Tian, W. B.; Zhang, Y. M.; Sun, Z. M. In situ synthesis of CNTs@Ti_3C_2 hybrid structures by microwave irradiation for high-performance anodes in lithium ion batteries. *J. Mater. Chem. A* **2018**, *6,* 3543-3551.

14. Seo, D.; Kim, M.-R.; Kyu Song, J.; Kim, E.; Koo, J.; Kim, K.-C.; Han, H.; Lee, Y.; Won Ahn, C. Hollow Ti_3C_2 MXene/carbon nanofibers as an advanced anode material for lithium-ion batteries. *ChemElectroChem* **2022**, *9,* e202101344.

15. Rai, A. K.; Anh, L. T.; Gim, J.; Mathew, V.; Kang, J.; Paul, B. J.; Song, J.; Kim, J. Simple synthesis and particle size effects of TiO_2 nanoparticle anodes for rechargeable lithium ion batteries. *Electrochim. Acta* **2013**, *90,* 112-118.

16. Zhang, Z.; Yuan, X.; Peng, Y.; Zhao, S.; Zhou, N. First-principles calculations study of TiS_2/Ti_2CS_2 heterostructure as an anode material for Li/Na/K-ion batteries. *Comp. Mater. Sci.* **2022**, *215,* 111784.

17. Huang, X.; Tang, J.; Luo, B.; Knibbe, R.; Lin, T.; Hu, H.; Rana, M.; Hu, Y.; Zhu, X.; Gu, Q.; Wang, D.; Wang, L. Sandwich-like ultrathin TiS_2 nanosheets confined within N, S codoped porous carbon as an

effective polysulfide promoter in lithium-sulfur batteries. *Adv. Energy Mater.* **2019**, *9,* 1901872.
18. Lung-Hao Hu, B.; Wu, F.-Y.; Lin, C.-T.; Khlobystov, A. N.; Li, L.-J. Graphene-modified $LiFePO_4$ cathode for lithium ion battery beyond theoretical capacity. *Nat. Commun.* **2013**, *4,* 1687.
19. Naguib, M.; Halim, J.; Lu, J.; Cook, K. M.; Hultman, L.; Gogotsi, Y.; Barsoum, M. W. New two-dimensional niobium and vanadium carbides as promising materials for Li-ion batteries. *J. Am. Chem. Soc.* **2013**, *135,* 15966-15969.
